# Stego on FPGA: An IWT Approach

**DOI:** 10.1155/2014/192512

**Published:** 2014-02-26

**Authors:** Balakrishnan Ramalingam, Rengarajan Amirtharajan, John Bosco Balaguru Rayappan

**Affiliations:** School of Electrical & Electronics Engineering, SASTRA University, Thanjavur 613401, India

## Abstract

A reconfigurable hardware architecture for the implementation of integer wavelet transform (IWT) based adaptive random image steganography algorithm is proposed. The Haar-IWT was used to separate the subbands namely, LL, LH, HL, and HH, from 8 × 8 pixel blocks and the encrypted secret data is hidden in the LH, HL, and HH blocks using Moore and Hilbert space filling curve (SFC) scan patterns. Either Moore or Hilbert SFC was chosen for hiding the encrypted data in LH, HL, and HH coefficients, whichever produces the lowest mean square error (MSE) and the highest peak signal-to-noise ratio (PSNR). The fixated random walk's verdict of all blocks is registered which is nothing but the furtive key. Our system took 1.6 µs for embedding the data in coefficient blocks and consumed 34% of the logic elements, 22% of the dedicated logic register, and 2% of the embedded multiplier on Cyclone II field programmable gate array (FPGA).

## 1. Introduction

Cryptography [1–4] and steganography [5–9] are considered as the most prominent solutions among numerous techniques developed in the field of information security, particularly in all kinds of secure information (sensitive) systems to quash unauthorized attacks and protect the secret information during transmission and storage. In cryptography, the data is scrambled into an unreadable format prior to transmission or storage for hiding the contents from an attacker. The intended user accesses the data by unscrambling the data through the secret key which can be either a private or public key [1–4]. The drawback of the technique lies in the fact that it encrypts the data but does not hide its existence and the cryptic data can often entice the attackers [10]. Steganography on the other hand is a prowess of blotting out the secret content in a host medium without altering the properties of the latter with the intention that the veiled message is unperceivable (except for the receiver) [10]. Among different kinds of hosting medium namely image, audio, video, and so forth, image is considered as the most promising among the cover media due to the fact that it is easy to obtain with reasonable hiding capacity and distortion tolerance [10]. In comparison with cryptography, steganography caters a privileged echelon of privacy and security as it makes the secret information altogether invisible.

In steganography technique the secluded text is driven in cover files in two ways such as spatial domain [11–17] and transformed domain [18–25]. In the former, data gets buried in a cover's pixels using various algorithms such as least significant bit (LSB) [11–15], LSB using random scans [15], pixel indicator [16, 17], and pixel value differencing (PVD) [17]. These techniques are widely being used by researchers to achieve stego objects with better imperceptibility. Discrete-Cosine Transform (DCT) [18–20] and wavelet transform [21–24] are widely used for data hiding. In this technique, transformed coefficients are responsible of the covert content. The advantage of embedding in the transform domain is robustness, that is, the ability to withstand modification in the image such as rotations or cropping. In wavelet based data hiding, integer wavelet transform (IWT) [21–23] based data hiding has given high payload and better PSNR and MSE to stego object than discrete wavelet transform (DWT) [24] based data hiding because floating point coefficient creates a problem for high data hiding and gives not as much of PSNR.

There are works in steganography through FPGA [25–29], but they are in spatial domain [27–29]. This work advises the reconfigurable hardware for adaptive integer wavelet based data hiding which embeds the large amount of data in random scan technique to improve complexity and also give high PSNR and good payload. This paper is organized as follows. The necessary introduction for IWT is given in Section 2.  Section 3 describes the proposed FPGA steganography methodology using SFC in IWT followed by the hardware implementation in Section 4.  Section 5 explores hardware synthesis and performance analysis. The results and discussion are given in Section 6. Finally the conclusion is given in Section 7.

## 2. Integer Wavelet Transform

This paper habituates Haar IWT to infix secret bit stream in the cover file (image). As this is the case, IWT winds up with high and low coefficients of frequency in cover. The former is gained through flanking pixel pairs' edge information, whereas the latter is gained through stifling the same in all pixels.

First stage IWT is as follows:

(1) H=Co−Ce,L=Ce+⌊H2⌋,

where *C*_*o*_ = pixels in odd columns and *C*_*e*_ = pixels in even columns.

Consequently, this first stage leads to the next stage processes that involve high pass and low pass filter banks to find IWT coefficients. It results in four sub bands (LL, LH, HL and HH) out of which LL sub band has highly sensitive information. The rest of the bands have the in depth cover information.

Second stage IWT is as follows:

(2) LH⁡=Lodd−Leven,LL=Leven+[LH⁡2],HL=Hodd−Heven,HH=Heven+[HL2].

In the second stage, H_odd_ = H band's odd row, L_odd_ = L band's odd row, H_even_ = H band's even row, and L_even_ = L band's even row.

The confidential message bits are rooted in wavelet coefficients. Inverse IWT is exercised in the ensuing coefficients to get stego output and this can be used for further communication. Since IWT encourages reversible makeover, at the receiving end, secret bit stream is revived with the help of the same secret key applied to the transmitter.

## 3. Proposed Method

**Figure 1:**
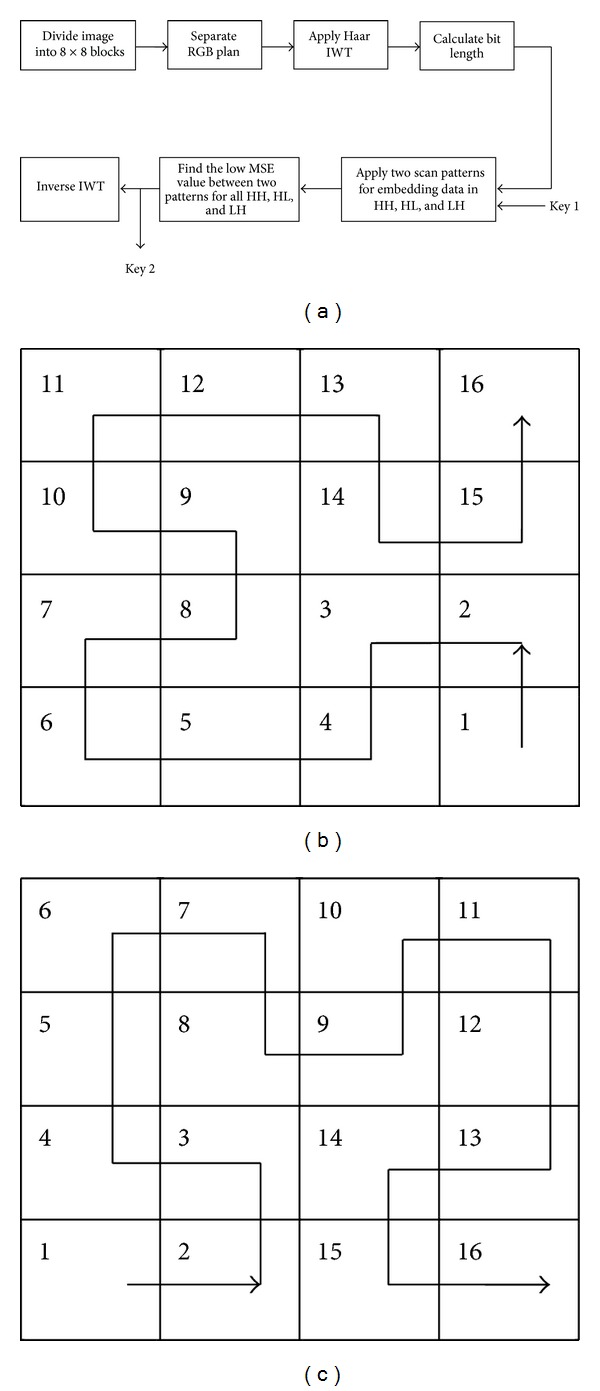
(a) Adaptive IWT block diagram, (b) Moore SFC, and (c) Hilbert SFC.

Schematic diagram for this proffer was publicized in Figure 1(a). IWT is employed to obtain wavelet coefficients for burying the secret message. Key 1 ranges from numbers one to four deciding the total bits to be infixed in cover file and by varying its increased capacity it can be attained. Randomized embedding of encrypted secret bits was done through SFC patterns [15], namely, Hilbert and Moore, which are shown in Figures 1(b) and 1(c) in HH, HL, and LH bands of every 4 × 4 coefficient block. For apiece traversing trails, least MSE and utmost PSNR were computed and the one which gave the best result was chosen for final embedding. Two separate keys, 00 (Moore) and 01 (Hilbert), were assigned for the two paths; for every block, a key was set according to the best path.

### 3.1. Algorithm

1. Read the cover image of size 128 × 128 × 3 and secret data.
2. Divide the image into 8 × 8 blocks.
3. Divide them into red, blue, and green planes.
4. Choose one block using pseudorandom number generator.
5. Apply Haar wavelet transform to the randomly selected block to form subbands.
6. Calculate bit length to estimate the embedding capacity of each coefficient.
7. Assign key 1 for *k*-bit embedding.
8. Assign key values for the two scan patterns. Let it be key 2.
9. For every 8 × 8 coefficient, apply the two scan patterns and determine MSE in each plane for every pattern.
10. Select the minimum MSE value between two patterns and using that particular pattern, embed the secret data by LSB substitution using *k*-bit embedding.
11. Take inverse IWT to reproduce the stego block
12. Repeat the process till the last bit of secret content gets entrenched.
13. Store the result as stego image.
14. Communicate the two keys to the receiver.

## 4. IWT Hardware Implementation

**Figure 2:**
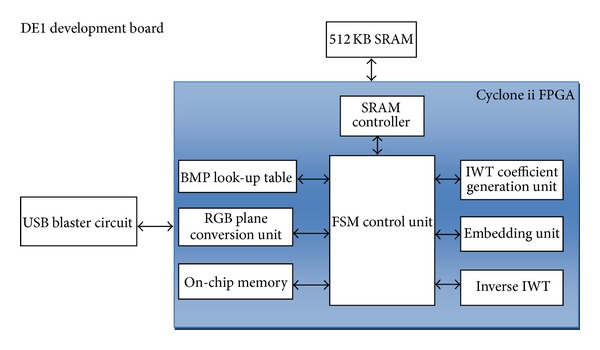
Block diagram of IWT reconfigurable hardware stego processor.

The proposed IWT based data hiding architecture is shown in Figure 2. The design comprises of the following major blocks in FPGA architecture; finite state machine based control unit, address generation unit, SRAM controller, on chip memory, IWT coefficient generation unit, embedding unit and Mean Square Error Module.

### 4.1. Finite State Machine (FSM) Control Unit

**Figure 3:**
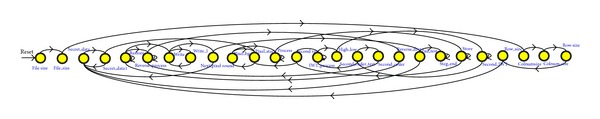
IWT based data hiding state diagram representation.

The state diagram of FSM control unit is shown in Figure 3. This controls the address generator module, SRAM controller, IWT coefficient generation unit, data embedding unit, inverse IWT, and embedding block. FSM consists of the following status registers which hold the current state and the next state of the process, pixel counter that counts the number of processed pixels, message counter that counts the embedded message bits, row address counter that counts the number of processed rows, column address counter to count the number of processed columns, and block counter for counting the processed *M* × *N* blocks, and column and row pointers hold the current column and row address. Memory pointer directs the address generator to the next memory location from where it is to receive pixel data and encrypt message bit.

### 4.2. Address Generator

**Figure 4:**
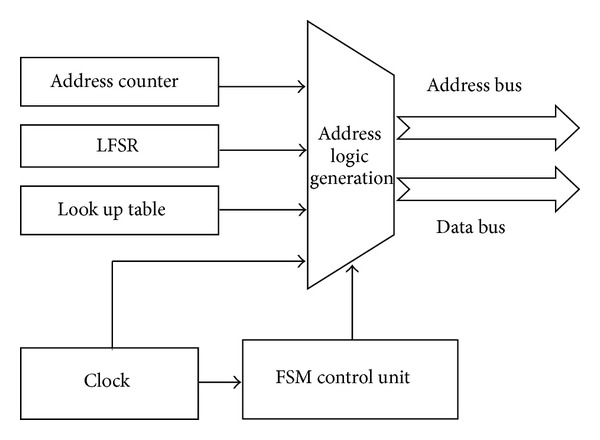
Hardware model of address generation unit.

The hardware model of address generator is shown in Figure 4. It generates address for SRAM controller to read the pixel value and encrypted message. It consists of address counter, linear feedback shift registers, pattern lookup table, and BMP header lookup table. LFSR is the combination of sequential shift, register and feedback logic. The address counter is a simple counter that generates the memory address to read the pixel value from SRAM. BMP header lookup table was used to read the header file information (Table 1) from BMP image file stored in external SRAM and copy it into internal cache memory. This header file information was used to know the image's dimensions. LFSR engenders random sequence for user's given value to choose one *M* × *N* pixel block among an *N* number of blocks. Also the same sequence is generated at the recipient side.

### 4.3. SRAM Controller

**Figure 5:**
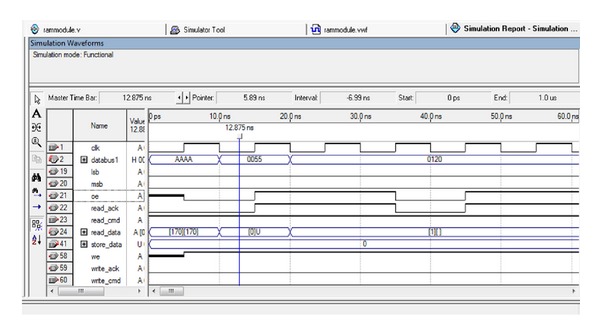
SRAM *reads *timing diagram.

**Figure 6:**
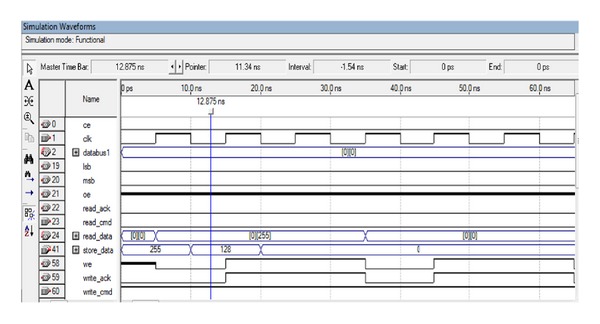
SRAM *writes *timing diagrams.

The SRAM controller communicates with the 256 K × 16 asynchronous CMOS static RAM (SRAM) chip on ALTERA DE2 board. The SRAM controller enables users to read or write the SRAM from a master device (such as the FPGA) as a normal memory operation. For 8-bit or 16-bit data, there will be, respectively, 2 clock cycles and 1 clock cycle of latent period for read and write operations. It has 16 bit data bus, 18 bit address bus, and three control signals for read and write operations and one for word or byte mode selection. Timing diagram of SRAM is shown in Figures 5 and 6. The SRAM controller supports a clock frequency of 50 MHz.

### 4.4. On-Chip Embedded Memory

**Figure 7:**
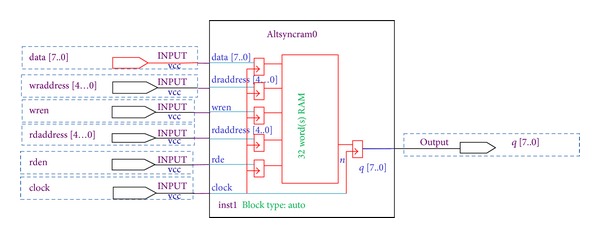
On-chip embedded memory.

The FPGA embedded memory presented in Figure 7 contains columns of M4K memory blocks to configure as on-chip memory for storing *M* × *N* pixel values, encrypted secret, and *M* × *N* stego pixels in addition to RGB plane values. Simple dual-port mode abides concurrent read and write operations. Here, the memory blocks possess one write enable and one read enable signal. The above illustrated waveform is the result of the design's normal read conditions. The read occurs at the mounting edge of the enabled clock cycle. To read in simple dual port mode, *Read enable *port is ought to be enabled.

### 4.5. IWT Coefficient Generation Block

**Figure 8:**
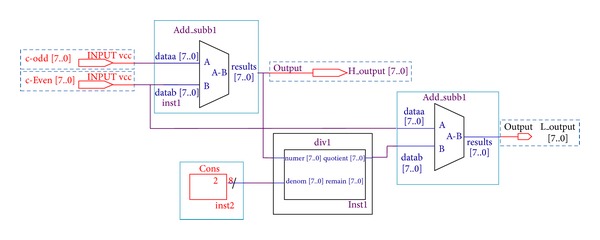
RTL view of IWT coefficient generation.

Register transfer level (RTL) view of coefficient generation blocks is shown in Figure 8. It is comprised of two libraries of parameterized modules (LPM) such as LPM subtraction modules and LPM divider module. These LPM modules render all the three planes coefficients separately and store them on chip memory modules.

### 4.6. Embedding Block

**Figure 9:**
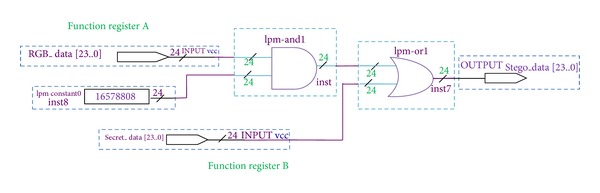
Functional block diagram of embedding module.

The embedding module's functional diagram is given in Figure 9. It inhabits function registers A, B along with cascaded AND - OR logic modules of 24 bits wide each. The former is useful in storing secret message bits and 2D IWT coefficients during substitution process. After enshrouding the data into 2D IWT coefficient values, they get laid in inverse IWT Block. This block rejuvenates the pixels from 2D IWT coefficient values. The same functional diagram is pertinent to the inverse process.

### 4.7. Mean Square Error Module

**Figure 10:**
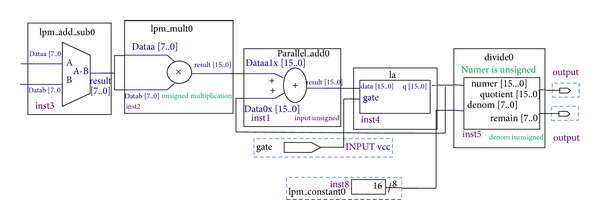
Mean square error hardware model.

The MSE hardware is the collective squared error between the original and stego images. Hardware model of MSE is shown in Figure 10; it consists of mega function LPM_ADD_SUB, LPM multiplier, latch, and divider. LPM_ADD_SUB unit produces an output containing the difference of the input values, and the LPM _MULT unit carries out the square root functions; it squares the difference value of LPM_ADD_SUB unit. The parallel adder unit carries out the summation process by summing the squared difference value with previous difference value; latch is used to store the summing output and its output is fed back into one of the inputs of parallel adder. Divider unit divides the summation output with *M* × *N* value and produces MSE result.

## 5. Hardware Synthesis and Performance Analysis Results

**Figure 11:**
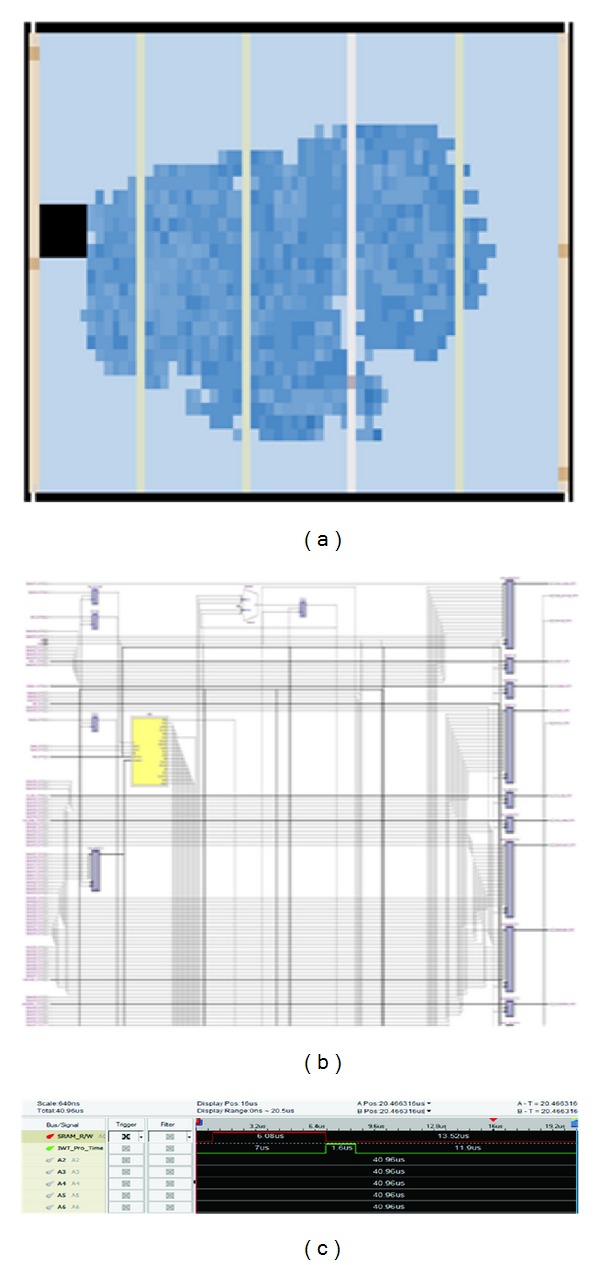
(a) Chip planner view, (b) RTL view, and (c) logic analyzer timing output.

The two-dimensional IWT reconfigurable stego processor architecture was developed using IEEE standard Verilog HDL and is trialed on Cyclone II EP2C35F672C6 FPGA. Its compilation report is shown in Table 2. The design consumes 34% of the logic elements, 22% of the dedicated logic registers, and 2% of the embedded multipliers of a Cyclone II FPGA. The end results for RTL view and Chip planner are shown in Figures 11(a) and 11(b). Time taken for 2D IWT coefficient and data embedding in coefficient was calculated with the help of zero plus logic analyzer tool and the results are shown in Figure 11(c).

The implemented algorithm consumed 1.6 *μ*s for IWT-coefficient generation, embedding the data in coefficients and MSE calculation. It took 6.08 *μ*s to read 8 × 8 blocks and RGB separation.

## 6. Results and Discussions

**Figure 12:**
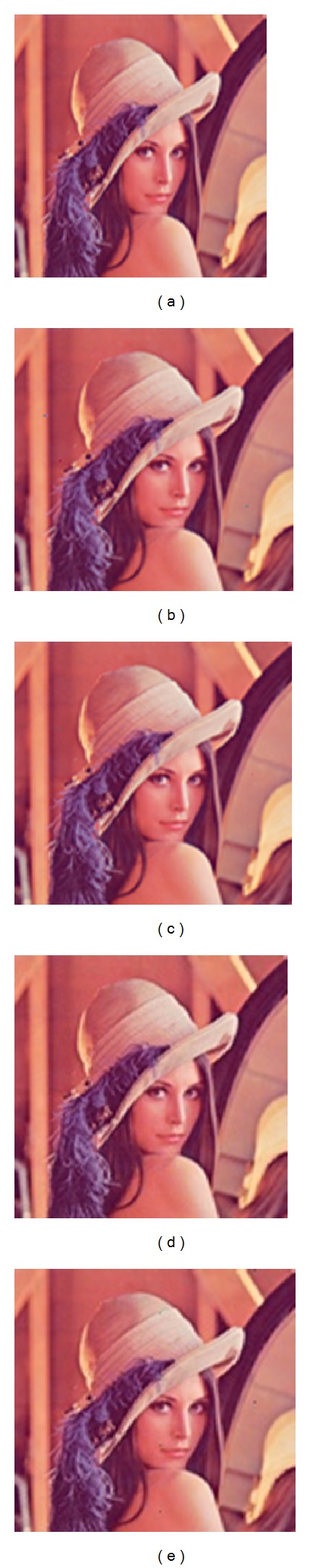
(a) Cover image Lena, (b) adaptive IWT, (c) adaptive spatial technique, (d) Moore, and (e) Hilbert.

**Figure 13:**
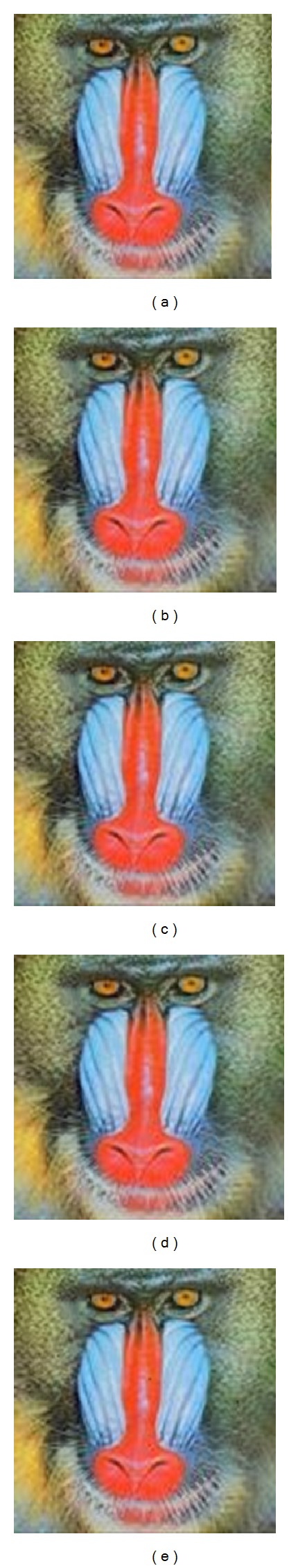
(a) Cover image baboon, (b) adaptive IWT (c), adaptive spatial technique, (d) Moore, and (e) Hilbert.

In this effectuation, color digital images Lena and Baboon of dimension 128 × 128 were chosen as covers, as in Figure 12(a) and Figure 13(a). This work was vindicated through MSE and PSNR:

(3) $$\begin{array}{c} \text{MSE}=\frac{1}{MN}{\sum_{i=1}^{m=1}\sum_{j=1}^{n=1}{\left({X_{ij}}_{}-{Y_{ij}}_{}\right)}^{2}}^{}. \end{array}$$

Here *M* and *N* stand for the number of pixels in horizontal and vertical dimensions of cover file (image); *X*_*i*,*j*_ and *Y*_*i*,*j*_ give the number of pixels in original and stego image accordingly. PSNR is

(4) $$\begin{array}{c} \text{PSNR}=10\;\text{lo}{\text{g}}_{10}\left(\frac{I_{\max}^{2}}{\text{MSE}}\right)dB. \end{array}$$

In this analysis key-2 was used to find the low MSE scan pattern for random embedding of the data in coefficients.  Table 3 shows comparison of the proposed system with other spatial techniques (Moore, Hilbert, and adaptive random spatial data hiding technique) and its output stego images are shown in Figures 12(b)–12(e) and 13(b)–13(e). From the table it is vivid that adaptive IWT technique provides high PSNR and low MSE for *k* = 1–3 bit embedding.

## 7. Conclusion

This study exhibits an adaptive integer wavelet transform based data hiding plot rendering soaring payload simultaneously asseverating absolute stego-image visual quality. When likened with the available literature, PSNR is increased in this system with intelligent use of key-1 and key-2. Moreover, these keys not only provide high security but also increase the capacity. The main drawback of the IWT based data hiding is the computational overhead but this present implementation overcomes this problem, using field programmable gate arrays (FPGA) which provides high speed implementation because of parallelism. This work is currently being extended to develop a consecrated stego processor by means of FPGA chip.

## Figures and Tables

**Table 1:** Structure of BMP header.

Address of header function (Windows OS)	Function	Total number of bytes
00-01	Header (BM)	Two bytes
02–05	Image file size	Four bytes
06–09	Reserved bytes	Four bytes
10–13	Starting address of pixel data	Four bytes
14–17	Size of data header	Four bytes
18–21	Image width (row)	Four bytes
22–25	Image height (column)	Four bytes
26-27	Number of colour planes used (always 1)	Two bytes
28-29	Number bits per pixel	Two bytes
30–33	Compression method being used	Four bytes
34–37	Size of stored pixel data	Four bytes
38–41	Width resolution	Four bytes
42–45	Height resolution	Four bytes
46–49	Number of colours actually used	Four bytes
50–53	Number of important colours (0 = all colours important)	Four bytes
54	Beginning of the pixel array	Four bytes

**Table 2:** IWT stego processor compilation report.

Family	Cyclone II
Device	EP2C35F672C6
Timing models	Final
Met timing requirements	Yes
Total logic elements	11.222/33.216 (34%)
Total combinational functions	8.792/33.216 (26%)
Dedicated logic registers	7.412/33.216 (22%)
Total registers	7412
Total pins	44/475 (9%)
Total virtual pins	0
Total memory bits	0/483.840 (0%)
Embedded multiplier 9-bit elements	2/70 (3%)
Total PLLs	0/4 (0%)

**Table 3:** Comparison of MSE and PSNR values of adaptive IWT approach with the spatial data hiding approach.

Cover image	Method		MSE	PSNR	MSE	PSNR	MSE	PSNR
			*k* = 1		*k* = 2		*k* = 3	
LENA	IWT [HH, HL, LH]	R	0.0417	61.9329	0.0781	59.2024	0.2448	54.2426
		G	0.0521	60.9638	0.1094	57.7416	0.3538	52.6432
		B	0.0313	63.1823	0.1146	57.5396	0.2966	53.4090
	Moore	R	0.3945	52.1703	3.1057	43.2092	4.0354	40.3233
		G	0.2143	54.8207	0.9307	48.4427	3.8205	42.3096
		B	0.3424	52.7854	1.0490	47.9232	4.5052	41.5936
	Hilbert	R	0.2541	54.0806	2.4028	44.3236	5.7147	40.5613
		G	0.2527	54.1043	0.9184	48.5007	4.6304	41.4746
		B	0.4841	51.2815	0.8955	48.6100	5.3166	40.8745
	AR	R	0.2524	54.0954	1.0687	47.8424	3.7331	42.4101
		G	0.2139	54.8285	0.8689	48.7409	3.7019	42.4466
		B	0.1664	55.9197	0.8599	48.7864	3.9633	42.1503

BABOON	IWT [HH, HL, LH]	R	0.0417	61.9329	0.1094	57.7416	0.3490	52.7031
		G	0.0521	61.9638	0.0885	58.6593	0.4567	51.5344
		B	0.0521	61.9638	0.7965	49.1189	0.9244	48.4722
	Moore	R	0.4244	51.8535	2.4705	44.2093	5.3617	40.2543
		G	0.8569	48.8014	1.5601	46.1993	4.2309	41.8665
		B	0.6943	49.7155	0.9703	48.2619	4.1827	41.9163
	Hilbert	R	0.6796	49.8082	2.2251	44.6574	4.8661	41.2589
		G	0.8629	48.7711	1.3373	46.8684	4.5632	41.5381
		B	0.2593	53.9935	0.9038	48.5698	4.9245	41.2071
	AR	R	0.2978	53.3920	1.0556	47.8960	3.7609	42.3778
		G	0.1864	55.4273	0.8581	48.7954	3.6576	42.4984
		B	0.1673	55.8970	0.8510	48.8313	4.0222	42.0862
